# The Therapeutic Value of Hope

**Published:** 1909-05-08

**Authors:** 


### The Hospital

A Journal of

The Medical Sciences and Hospital Administration.

New Series. No. 114, Vol. V. [No. 1186, Vol. XLVI.] SATURDAY, MAY 8, 1909.

### The Therapeutic Value of Hope.

Of the qualities which make for success in the practice of medicine, using the word in its most comprehensive sense, it is recognised that none is more vital than the faculty of inspiring hope. We are ready enough to accept the truism, but in daily life many of us treat it cavalierly, as a pious resolution which it is respectable to profess but tiresome to materialise in conduct. Nevertheless, the practice of optimism is far better able to stand alone than is the pure practice of technical healing. The inveterate optimist whose technical knowledge is minimal will do more good in the world than his professional brother who stultifies an immeasurably superior technical ability by exercising it with an air of gloom.

It is something of a blot upon us that our textbooks of materia medica, which dwell lovingly upon pharmacological excitants and depressants, have never a word to say about the somatic influences of the emotions. Yet these agents are unceasingly operative, and may be so potent as to neutralise, to no small extent, the effect of their pharmacological opposites. For example, it needs a larger dose of a narcotic to quiet an individual labouring under intense mental excitement than would suffice for the purpose were he in a state of composure; and a man under circumstances of mental stress can take with impunity a quantity of alcohol which ordinarily would leave him maudlin.

These are common experiences, and the wonder is that the lessons they teach us are not more formally urged. The neglect is probably due to the vagueness of the subject. Medicine, having wallowed for centuries in vagueness and superstition, is naturally shy of risking a repetition of the experience; indeed, until the relations of mind and body are more precisely established, it is likely that she will continue to look askance, in practice if not in theory, upon those who insist upon the importance of the relation, and the necessity for regarding the mind as a potential item of the pharmacopœia. But the clearing of the ground has begun, for has not Pawlow given us chapter and verse for the psychic secretion of the stomach? We must look to experimental physiology to give us more such demonstrations. Without them prophets may cry that the functions of mind and body are indissolubly merged, but they will continue, as heretofore, to cry in the wilderness.

The capacity for diffusing hope is not given to all equally. Some are temperamentally so endowed that their gaze falls instinctively upon the brighter elements in any conjunction of circumstances, to the exclusion of the more sombre. This mental equipment evolves the type of medical man commoner on the stage than in real life, yet not entirely fictitious. He strides, beaming, into the sick-room, and, if it is physically possible, slaps the patient on the back. If the back is pre-occupied by pillows or a mattress, he proceeds verbally in the same direction, endeavouring by a gust of jovial talk and banter to dissipate the greyness of atmosphere which so readily envelopes a sick-room. Often enough his robust cheeriness proves infectious, and leaves the patient with a sense of well-being which the cleverest combination of drugs is powerless to impart. But the danger which threatens such a practitioner lies in a lack of intuitive delicacy, of tact. This defect is a common, if not essential, accompaniment of the cheeriness and *bonhomie* which characterise the type whose explosive joviality represents an absolutely natural overflow of animal spirits. For those who command such blessings, in abundance and continually, are proportionately incapacitated from seeing with the peevish eye of an invalid. Thus it happens, on exceptional occasions, that the cheery and good-natured doctor is written down an uncouth and callous boor. For this reason the most uniformly successful disseminator of hope is he who practises optimism not because he is blind to all other considerations, but because contemplation and experience have persuaded him of its remedial power. Such a man, recognising that his demeanour is a therapeutic asset, capable on occasion of transcending the virtues of his strychnia and bromides, will temper his geniality to the humour of the patient, and graduate his doses of it, *secundum artem*. Not infrequently he will find himself called upon to display hopes he does not entertain—a piece of counterfeit which, if effectually disguised, may be a triumph of therapeutics, though a superficial judgment might give it an ugly name.

In the department of prognosis among private patients the young practitioner will find a special merit attaching to optimism. Most men take with them from the schools the impress of their hospital experience in prognosis—a most fallacious guide, for hospital patients are drawn from the most reduced and least resistant ranks of humanity; they are often worn by hardship and want, or debilitated by excesses, and are always incapable of commanding the ameliorations which the power of the purse places within the reach of the better-to-do. Swayed by their past associations, young men may easily enter practice, convinced that albuminuria in a man past middle age is tantamount to a sentence of death to be carried into effect in a year or two. They will be lucky if they do not hint their fears, for it is no joke to have to face, over a period of 15 or 20 years, a man whose impending demise one has regretfully predicted. It is said of a prominent member of our profession that the sufferer from heart disease, phthsis, or what not, leaves his consulting-room in a frame of mind approaching exultation that he should have been so particularly favoured as to contract the pleasant malady in question. The faculty thus humorously expressed of promoting good cheer in the hearts of sick men is equally precious to the doctor and the patient, and cannot be too sedulously cultivated.

